# Molecular and Cellular Aspects of Rhabdovirus Entry

**DOI:** 10.3390/v4010117

**Published:** 2012-01-18

**Authors:** Aurélie A. V. Albertini, Eduard Baquero, Anna Ferlin, Yves Gaudin

**Affiliations:** Laboratoire de Virologie Moléculaire et Structurale, Centre de Recherche de Gif, CNRS (UPR 3296), Avenue de la Terrasse, 91198, Gif sur Yvette Cedex, France; Email: alberti@vms.cnrs-gif.fr (A.A.V.A.); baquero@vms.cnrs-gif.fr (E.B.); ferlin@vms.cnrs-gif.fr (A.F.)

**Keywords:** rhabdovirus, rabies virus, vesicular stomatitis virus, endocytosis, membrane fusion, glycoprotein

## Abstract

Rhabdoviruses enter the cell via the endocytic pathway and subsequently fuse with a cellular membrane within the acidic environment of the endosome. Both receptor recognition and membrane fusion are mediated by a single transmembrane viral glycoprotein (G). Fusion is triggered via a low-pH induced structural rearrangement. G is an atypical fusion protein as there is a pH-dependent equilibrium between its pre- and post-fusion conformations. The elucidation of the atomic structures of these two conformations for the vesicular stomatitis virus (VSV) G has revealed that it is different from the previously characterized class I and class II fusion proteins. In this review, the pre- and post-fusion VSV G structures are presented in detail demonstrating that G combines the features of the class I and class II fusion proteins. In addition to these similarities, these G structures also reveal some particularities that expand our understanding of the working of fusion machineries. Combined with data from recent studies that revealed the cellular aspects of the initial stages of rhabdovirus infection, all these data give an integrated view of the entry pathway of rhabdoviruses into their host cell.

## 1. Introduction

The Rhabdoviridae family is grouped in the order Mononegavirales together with the Filoviridae (e.g., Ebola Virus), the Paramyxoviridae (e.g., measles and respiratory syncytial viruses) and the Bornaviridae (e.g., Borna disease virus). All of these viruses are enveloped and have a non-segmented genome made of a single stranded negative-sense RNA molecule. 

Among Mononegavirales, Rhabdoviruses have the most diverse hosts. They are widespread among a great diversity of organisms such as plants, insects, fishes, mammals, reptiles and crustaceans [1]. This family has a long history and it has been recently shown that the genomes of several arthropods contain numerous integrated elements from Rhabdoviruses with some integration events that are at least 11 million years old [2]. 

Based on their structural properties, antigenicity and phylogenetic analyses, Rhabdoviruses have been grouped into six genera. Lyssavirus (prototype: rabies virus—RABV) and Vesiculovirus (prototype: vesicular stomatitis virus—VSV) are the two best studied genera. Other genera include the Ephemerovirus genus (prototype: bovine ephemeral fever virus), the Novirhabdovirus genus (which contains many fish viruses such as the infectious hematopoietic necrosis virus) and two genera that are arthropod-borne and infect plants: Cytorhabdoviruses (prototype: Lettuce necrotic yellows virus) and Nucleorhabdoviruses (prototype: Potato yellow dwarf virus). In addition, numerous identified rhabdoviruses are still unclassified. 

All rhabdoviruses have a rigid bullet shape with a flat base and a round tip. The genome of rhabdoviruses comprises up to ten genes among which only five are common to all members of the family. These genes encode the nucleoprotein (N), the phosphoprotein (P), the matrix protein (M), the glycoprotein (G) and the viral polymerase (L). The genome is associated with N, L and P to form the nucleocapsid, which is condensed by the matrix protein into a tightly coiled helical structure. The condensed nucleocapsid is surrounded by a lipid bilayer containing the viral glycoprotein G that constitutes the spikes that protrude from the viral surface. 

G plays a critical role during the initial steps of the infectious cycle. First, it recognizes receptors at the viral surface and after virion endocytosis, it mediates the fusion between the viral and the endosomal membranes.

## 2. Basic Biochemical Properties of the Rhabdovirus Glycoprotein

G is a type I membrane glycoprotein. After cleavage of the amino-terminal signal peptide, the complete mature glycoprotein is approximately 500 amino acids long (495 for VSV and 505 for RABV). The bulk of the mass of G is located outside the viral membrane and constitutes the amino‑terminal ectodomain (452 for VSV and 440 for RABV). G is anchored in the membrane by a single α-helical transmembrane (TM) segment. The small intraviral domain is likely involved in interactions with internal proteins and there is evidence for RABV G interaction with M protein [3]. 

For both VSV and RABV, the glycoproteins form trimers [4,5,6,7,8]. This oligomeric organization is not stable at high pH and is sensitive to detergent solubilization [5]. In the case of VSV, there exists a dynamic equilibrium between monomers and trimers of G, both *in vitro* after detergent solubilization [9,10] and *in vivo* in the endoplasmic reticulum [11].

The rhabdovirus glycoprotein is N-glycosylated. The number of glycosylation sites may vary from one virus to another. For both VSV G and RABV G, it has been shown that the oligosaccharide chains are required for proper folding of the protein at two different levels: first, they increase the solubility of the folding intermediates and second, they allow the interaction of these folding intermediates with calnexin and calreticulin, which are chaperones with lectin properties [12,13,14]. 

The G ectodomain is the target for neutralizing antibodies [15,16,17,18,19]. The major antigenic sites of both VSV G and RABV G have been characterized. In the case of RABV, several hundred monoclonal antibodies (Mabs) have been used to characterize the antigenic structure of G. RABV G has two major antigenic sites: antigenic site II is located between amino-acids 34 and 42 and amino-acids 198 and 200 [17], and antigenic site III extends from amino acid 330 to 338 [18]. This latter site is associated with virulence and the replacement of arginine 333 by any other amino acid (except lysine) leads to an attenuated phenotype [18,20,21]. In addition to these major antigenic sites, one minor antigenic site and a few isolated epitopes have been described [22,23,24,25,26].

## 3. Rhabdovirus Receptors

VSV has a wide host spectrum: It infects both vertebrates and insects cells. Therefore, its receptor is a rather ubiquitous molecule. Phosphatidylserine (PS) has long been considered to be a viral receptor [27] despite the fact that it is only present at the surface of apoptotic cells. Recent results indicate that PS is not a receptor for VSV [28]. Other studies have suggested that gangliosides might play the role of the receptors in CER (chicken embryo related) cells [29]. Moreover, recent work has demonstrated that the endoplasmic reticulum chaperone gp96 is essential for infection with VSV [30]. Cells without gp96 or with catalytically inactive gp96 do not bind VSV-G. From these data, it has been proposed that gp96 is essential for the occurrence of functional VSV-G receptors at the cell surface, most likely because it is required for correct folding of either a proteinaceous receptor or an enzyme required for the synthesis of a glycolipid receptor [30].

In the case of RABV, apart from the very beginning and end of the infectious process, non-adapted isolates exclusively multiply and propagate in neurons, and *in vitro*, they can only infect established cell lines of neuronal origin. Several passages are required to select a fixed strain that is adapted to the multiplication in non-neuronal cell lines (such as BHK21 and Vero cells) [31,32,33]. Most of the fixed RABV laboratory strains have resulted from such an adaptation process. Although they have kept their neurotropism and propagate in the nervous system like street viruses, they have also acquired the ability to use ubiquitous receptors that are present at the surface of non-neuronal cell types [34]. As a consequence, among the many molecules that have been proposed to be RABV receptors [35], it is not clear which are actually used by natural isolates during animal infection.

Host cell treatment with different phospholipases has been shown to reduce the binding of fixed RABV strains suggesting that some phospholipids can play the role of viral receptors [36]. Similarly, cells pretreated with neuraminidase were shown to be non-susceptible to viral infection [37]. After incorporation of exogenous gangliosides such as GT1b and GQ1b, the cells recovered their susceptibility to RABV infection [38]. These results indicate that highly sialylated gangliosides are part of the cellular membrane receptor structure for the attachment of fixed RABV strains. 

In addition to phospholipids and gangliosides, three proteins have been proposed to play the role of viral receptors. Some evidence indicates that the nicotinic acetylcholine receptor (nAChR) acts as a RABV receptor [39]. First, a segment of RABV G has a sequence similarity to the snake venom curaremimetic neurotoxins which are potent ligands of nAChR [40]. Second, an interaction between RABV and purified Torpedo nAChR was demonstrated [41]. Finally, purified RABV was shown to bind the α subunit of nAChR in an overlay assay [42]. However, there is no direct evidence in animal models that this molecule is a RABV receptor. Furthermore, RABV can infect neurons that do not express nAChR [43], and nAChR is located mainly on muscle cells. Thus, although nAChR could account for the ability of street RABV to multiply locally in myotubes at the site of inoculation [44], which would facilitate the subsequent penetration into neurons, other molecules are needed to mediate viral entry into neurons.

The second protein that has been proposed to play the role of a RABV receptor is the neural cell adhesion molecule (NCAM) [45]. Preincubation of RABV with soluble NCAM inhibited its ability to infect susceptible cells. Moreover, the transfection of resistant L fibroblasts with the NCAM-encoding gene induces RABV susceptibility. Additionally, the infection of NCAM-deficient mice by RABV resulted in slightly delayed mortality and restricted brain invasion. This suggests that NCAM is a *bona fide* receptor *in vivo*.

The low-affinity nerve-growth factor receptor, p75NTR was identified as a ligand of a soluble form of RABV G [46]. The ability of RABV G to bind p75NTR was dependent on the presence of a lysine and an arginine in positions 330 and 333, respectively, which were known to control virus penetration into the motor and sensory neurons of adult mice [18,20,21]. Replacement of amino acids 318 and 352 were also shown to abolish interaction between p75NTR and RABV G [47]. Furthermore, p75NTR‑expressing BSR cells were permissive for a non-adapted fox RABV isolate (street virus). The glycoprotein from another genotype of lyssavirus (GT 6, European bat lyssavirus type 2) was also shown to bind p75NTR [48]. Nevertheless, mice lacking all the extracellular receptor domains were still susceptible to infection (of which the rate and specificity were unchanged), indicating that the RABVG-p75(NTR) interaction is not necessary for RABV infection of primary neurons [49].

Very little is known concerning the receptor of other rhabdoviruses, the only exception is the viral hemorrhagic septicemia virus (VHSV), a salmonid rhabdovirus, for which it has been shown that monoclonal antibodies (MAbs) directed against a fibronectin containing complex protect cells from the infection. Because the purified rainbow trout fibronectin was able to bind specifically to VHSV, fibronectin was proposed to be a receptor for VHSV and other fish rhabdoviruses [50].

## 4. Fusion Properties of Rhabdoviruses

After receptor binding, both RABV and VSV enter the cell by the endocytic pathway. The acidic environment of the endosomal compartment triggers a series of conformational changes of the viral glycoproteins that catalyze fusion between the viral and the endosomal membranes [51,52,53]. 

The pH dependence of fusion has been characterized for several rhabdoviruses [54,55,56]. Fusion is optimal around pH 6 and the threshold for fusion activity is approximately pH 6.5. 

As for other viruses that fuse at low pH, the exposure of the virion to low pH in the absence of a target membrane leads to the inactivation of the fusion properties of G [57,58,59]. Remarkably, unlike other viruses for which the low-pH induced fusion inactivation is irreversible [57], reincubation of the virus above pH 7 leads to the recovery of the initial fusion activity of rhabdoviruses [58,59]. 

Along with the fusion properties, it has been demonstrated that G can assume at least three different conformational states that have different biochemical properties [54,58]. The native pre-fusion state is observed at the viral surface above pH 7. In this conformation, G is thought to bind the viral receptors. Upon acidification, the virions are initially more hydrophobic [58], a feature that, in the absence of a target membrane, results in viral aggregation [59]. Using hydrophobic photolabeling, it has been demonstrated for both RABV and VSV, that G is then in an activated state that is able to interact with the target membrane as the first step of the fusion reaction [60]. After a longer incubation at low pH, the G post-fusion conformation is reached. In this conformation, the structure of G is antigenically distinct from both the pre-fusion and the activated conformation and, in electron microscopy, its ectodomain appears much more elongated than in the pre-fusion state (11 nm *versus* 8 nm) [58].

The structural transition is reversible and the pre-fusion state can be recovered from the post-fusion state by readjusting the pH above 7 [58]. In fact, there is a pH-dependent thermodynamic equilibrium between different states of G that is shifted toward the post-fusion state at low pH [61]. The transition toward the post-fusion state is highly cooperative upon proton binding: approximately 2.8 protons bind simultaneously to trimeric G to induce the conformational change [61].

This equilibrium explains why the low-pH induced inactivation is reversible. The reversibility of the conformational change is required to allow G to be transported through the acidic compartments of the Golgi apparatus and to recover its native pre-fusion state when incorporated in newly synthesized virions [62]. Other viruses, for which the fusogenic conformational change is irreversible (such as influenza virus, tick borne encephalitis virus or Semliki forest virus), have evolved different mechanisms to protect their fusion proteins from undergoing irreversible conformational changes in the Golgi apparatus [63]. For these viruses, it has been proposed that the high amount of energy released during the conformational change from the metastable pre-fusion state to the final stable post‑fusion state is used to form the high energy lipidic intermediates during the fusion reaction [64]. In the case of rhabdoviruses, the existence of an equilibrium between the different states implies that the energy released during the structural transition of a single trimer is small compared with the energetic barrier of the fusion reaction (the activation energy of the fusion process has been estimated to be in the range of 40 kcal/mol [54,65,66]). This indicates that a concerted action of several trimers is required. Indeed, for RABV, the minimal number of spikes involved in a fusion complex has been estimated to be approximately 15 [61]. 

The fusion pathway of rhabdoviruses with liposomes has been studied in detail. Neither RABV nor VSV require a specific lipid for fusion [59,67,68]. However, recent studies have indicated that the target membrane composition has an influence on the efficiency of the process. Particularly, it has been demonstrated that lipid bis(monoacylglycero)phosphate (BMP, also called lysobisphosphatidic acid), present in the internal vesicles of the endocytic pathway, favors VSV fusion when it is present in the target membrane [69]. 

An investigation of the effects of lipids with various dynamic molecular shapes on RABV-induced fusion has suggested that, similar to other enveloped viruses [70,71], RABV-induced fusion proceeds via the formation of an intermediate stalk that is a local lipidic connection between the outer leaflets of the fusing membranes [57]. A radial expansion of the stalk would induce the formation of a transient hemi-fusion diaphragm (*i.e.*, a local bilayer made by two initial inner leaflets) in which the formation of a pore and its enlargement would lead to complete fusion. It has been possible to trap and characterize an RABV fusion intermediate under suboptimal fusion conditions (pH slightly above the pH threshold for fusion and cold temperatures) [57]. This intermediate is apparently at a well-advanced stage of the fusion process when the hemi-fusion diaphragm is destabilized, but while lipid mixing is still restricted.

## 5. Structural Studies on VSV G

### 5.1. Crystallographic Structure of the Pre- and Post-Fusion States of the VSV G Ectodomain

**Figure 1:**
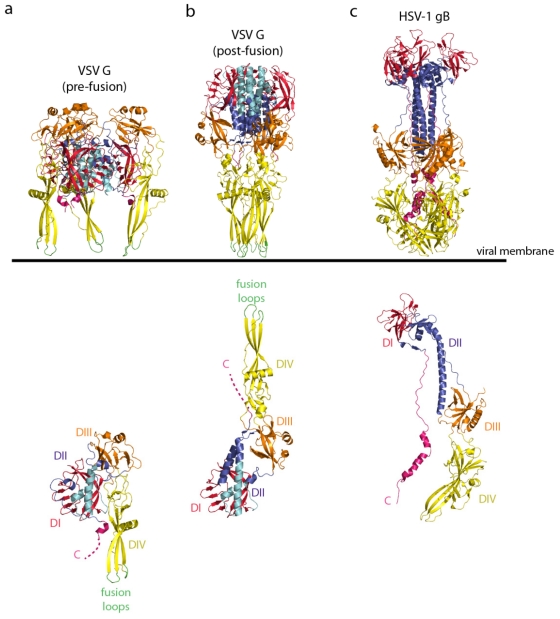
(**a**) Ribbon diagrams of the vesicular stomatitis virus (VSV) glycoprotein (G) pre-fusion trimer (top) and protomer (bottom) (PDB code = 2J6J [73]). (**b**) Ribbon diagram of the VSV G post-fusion trimer (top) and protomer (bottom) (PDB code = 2CMZ [72]). G is depicted by domains; the lateral domain (domain I) is in red, the pleckstrin homology domain (domain III) is in orange, and the fusion domain (domain IV) is in yellow. The trimerization domain is colored with two shades of blue (the part of G that retains its structure during refolding is in cyan, and the part of G that is refolded during the structural transition is in deep blue). The fusion loops are green and the C-terminus is pink. (**c**) Ribbon diagram of the HSV-1 gB trimer (top) and protomer (bottom) (PUB: 2GUM [74]). gB is colored by domains with the same color code as VSV G. The orientation of the VSV G and HSV-1 gB trimers toward the viral membrane is indicated. The VSV G protomers are aligned on the rigid block made of the lateral domain (in red) and the cyan part of the trimerization domain. The orientation of the post-fusion protomer is thus different in the top and the bottom of the figure.

The atomic structures of the pre- and post-fusion conformations of a soluble form of the VSV G ectodomain (G_th_, aa residues 1–422) have been recently determined [72,73] (Figure 1a,b). The soluble ectodomain had been generated by limited proteolysis of the viral particle with thermolysin. The structural organization of the two conformations of G appears to be very different from other viral fusion proteins described so far. However, amino acid sequence alignments revealed that all rhabdovirus glycoproteins share the same fold. 

Remarkably, the fold of G_th_ in its post-fusion state was the same as that of the HSV1 glycoprotein gB whose structure was published at the same time [74]. Together with the Epstein Barr virus glycoprotein gB [75] and the baculovirus glycoprotein gp64 [76] a new class of fusion proteins (class III) was defined (Figure 1c). VSV G is the only class III fusion protein for which the structures of both the pre- and post-fusion states have been determined by X-ray crystallography. The structures determined for other class III fusion glycoproteins are presumptive post-fusion conformations, based on their structural similarity with the post-fusion conformation of VSV G (Figure 1).

Four distinct domains of G_th_ have been identified: a β-sheet rich lateral domain (domain I in red), a central domain involved in the trimerization of the molecule (domain II, in blue), a pleckstrin homology domain (domain III, in orange) and a fusion domain (domain IV, in yellow). The organization of the VSV G fusion domain is similar (although not homologous) to that of the class II fusion proteins. It contains an extended β-sheet structure made of three β-strands that have two loops located at the tip that constitute the membrane-interacting motif of the G ectodomain. After the end of the trimerization domain (amino acid residue 405), 40 amino acids remain so that the polypeptide chain can reach the G TM segment but the structural organization is unknown after amino-acid residue 413 (resp. 410) for the pre-fusion domain (resp. post-fusion domain) (Figure 1a,b).

The major antigenic sites of RABV G are located in domain I (antigenic site III) and domain III (antigenic site II). Additionally, for RABV G, the p75NTR binding domain is located in lateral domain I and the putative binding site of nAChR is located in domain III. 

### 5.2. The Structural Transition

The G_th_ structures revealed that the conformational change from the pre- to post-fusion state involves a dramatic reorganization of the glycoprotein [51,73]. During the structural transition, domains I, III and IV keep their tertiary structure. However, they undergo rearrangements in their relative orientation due to secondary structure changes in the hinge region between the fusion (domain IV) and the pleckstrin homology domain (domain III) and due to a major refolding of the trimerization domain (domain II).

**Figure 2:**
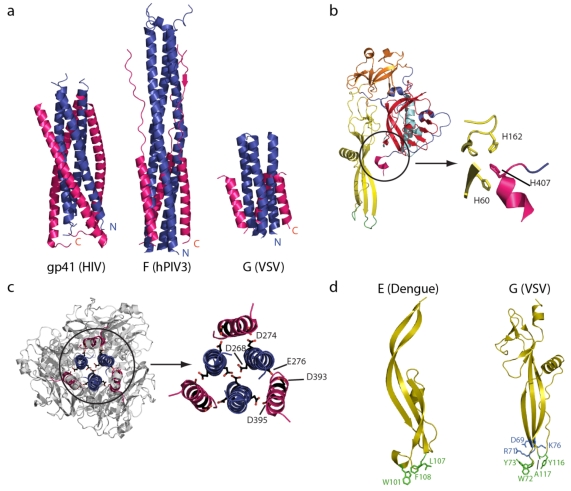
(**a**) A comparison of the central six-helix bundle of the class I viral fusion proteins (HIV gp41 and hPIV3 F) and class III viral fusion proteins (VSV G) in their post‑fusion conformation. The amino-terminal trimeric coiled-coil is in blue and the antiparallel lateral helices are in pink. (**b**) A close up view of the histidine cluster located at the fusion domain/C-terminal domain interface that has to be disrupted during the structural transition from pre- to post fusion structure. (**c**) Top view and close up of the VSV G trimerization domain showing the clusters of acidic residues in the post-fusion state. (**d**) The structural organization of the Dengue virus E fusion domain (left) and the VSV G fusion domain (right). The hydrophobic residues in the tips of the fusion domains are depicted in green and, for VSV G, charged residues that impede deep penetration of the fusion domain inside the membrane are in blue.

The refolding of G from pre- to post-fusion conformation is reminiscent of that of class I fusion protein such as the paramyxovirus fusion protein F [77] and the influenza hemagglutinin subunit 2 (HA2) [78]. As for class I, the pre- and post-fusion states are related by flipping both the fusion domain (domain IV) and the C-terminal segment relative to a rigid block composed of the lateral domain (domain I) and part of the trimerization domain (domain II) that retains its structure during molecule refolding. The reversal of the molecule around the rigid block involves the lengthening of the central helices (that form the trimeric central core of the post-fusion conformation) and the refolding of the three carboxy-terminal segments into helices that position themselves in the grooves of the central core in an anti-parallel manner to form a six helix bundle (Figure 1a,b). This organization is obviously very similar to that of the post-fusion hairpin structure of class I proteins. The G_th_ post-fusion trimer displays a six-helix bundle with the fusion domain at the amino-terminus of the central helices and the TM segments at the carboxy-terminus of the anti-parallel outer helices. The result is an elongated structure with the fusion domain and the TM segment at the same extremity of the molecule (Figure 2a). 

Both structures also allow the identification of amino acid residues playing the role of pH‑dependent molecular switches. In the pre-fusion state [73], there is a cluster of 3 conserved histidines (H60 and H162 in the fusion domain and H407 in the carboxy-terminal segment of the protein) (Figure 2b). At low pH, the protonation of these residues, by creating a cluster of positive charges, is likely to destabilize the interaction between the fusion domain and the C-terminal segment and might trigger the transition from the pre- to the post-fusion conformation. Indeed, it has been shown that chemical modification of histidines with diethylpyrocarbonate inhibits G fusion properties [79]. Reciprocally, in the post-fusion state, there are several acidic amino acids in the trimerization domain (domain II) that are sequestered close together [72] (Figure 2c). Sequence alignments reveal that this clustering is a conserved feature for all rhabdovirus glycoproteins [72]. In the VSV G post‑fusion conformation, these residues are protonated and form hydrogen bonds. Their deprotonation induces strong repulsive forces that destabilize the post-fusion trimer and triggers the conformational change toward the pre-fusion state. These residues are buried in the post-fusion conformation and are exposed to solvent in the pre‑fusion state. As a consequence, their pK_a_ is much higher in the post-fusion state than in the pre‑fusion state. This stronger affinity of the post-fusion state for protons explains the cooperativity of the rhabdovirus glycoprotein structural transition [61].

### 5.3. Interaction between G and Membranes

As mentioned above, the organization of the fusion domain resembles that of class II fusion proteins [80]. The main difference is that the membrane-interacting domain, located at the tip of the three-stranded β-sheet, is bipartite, made of two loops (Figure 2d). An alignment of the amino-acid sequences of several rhabdovirus glycoproteins reveals that these loops are only slightly hydrophobic but always contain polar aromatic residues (tryptophans and tyrosines). That these loops are indeed an essential part of the membrane interacting motif is consistent with mutagenesis experiments [81,82,83,84]. 

The presence of charged residues in the vicinity of the loops precludes any deep penetration of the fusion domain inside the target membrane. It is likely that the tryptophans and the tyrosines position themselves at the interface between the fatty acid chains and the head group layers of lipids [85] (Figure 2d). This interfacial interaction involving only a few residues cannot create a strong point of anchoring that can be used to pull the target membrane toward the viral one. Rather, this interaction likely facilitates the formation of point-like protrusions that have been proposed to be stalk precursors [86]. In addition, membrane deformation may be facilitated by the large number of spikes at the viral surface and their trimeric status, which allows multiple fusion loops to interact with the external leaflet of the target bilayer.

In the pre-fusion structure, unlike class I and class II fusion proteins, the fusion loops are not buried at an oligomeric interface and instead point toward the viral membrane [73]. It is therefore possible that in the pre-fusion conformation, the fusion loops also interact with the membrane. 

In addition to the fusion loops, two other domains have been demonstrated to play a major role in the fusion process. The first one is the TM segment. As for other viruses [87], replacement of VSV G TM segment by a glycophosphatidylinositol anchor results in the loss of the fusion properties of the protein [88]. Furthermore, mutagenesis studies have revealed a crucial role for the glycine residues of the TM segment at a late stage of the fusion process as mutations of these residues block fusion at the hemi-fusion stage [89]. 

The second domain is the membrane proximal domain [90]. A deletion of the 13 membrane-proximal amino acids reduces both the cell-cell fusion and the virus infectivity. The structure of this segment is not known but in the case of RABV G, it has been proposed to adopt an α-helical conformation with a strong amphipathic signature [91].

### 5.4. Cooperativity between Glycoproteins during the Fusion Process

**Figure 3:**
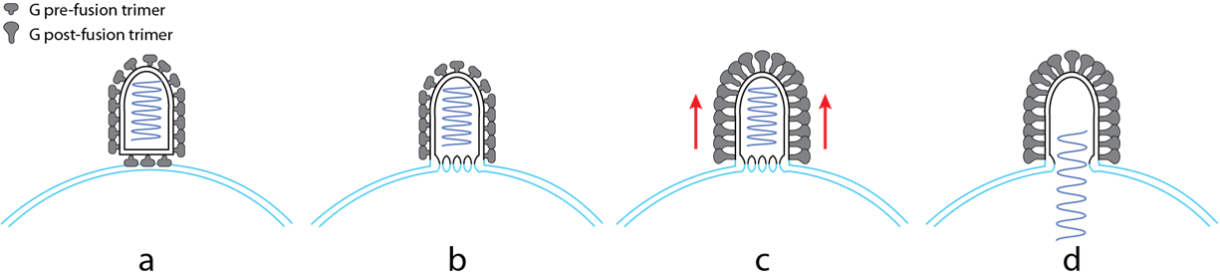
A model for VSV fusion [88]. In the first step (**a**), the flat base of VSV interacts with the target membrane most likely via activated glycoproteins that expose their fusion domain at their top. The local organization of G in this region may facilitate the formation of stalks and initial fusion pores (**b**). Then, a helical network of G in their post-fusion conformations is formed on the lateral part of the viral particle (**c**). The formation of this regular array likely induces tension in the membrane (red arrows) that drives pore enlargement and leads to complete fusion (**d**).

As mentioned above, for rhabdoviruses, the pH-dependent equilibrium between pre- and post-fusion conformations of G implies that a large number of spikes cooperate to achieve the fusion reaction [61]. Some recent data have provided new insights into the organization of the fusion machinery and suggest that distinct assemblies of fusion glycoproteins are involved at distinct stages of the fusion process [92] (Figure 3).

For RABV, a local hexagonal lattice of G was observed at the surface of some RABV mutants with a mutation in G affecting the kinetics of the structural transition when incubated under suboptimal fusion conditions [93]. Each hexagon was made up of 6 G trimers. Remarkably, a similar organization of G_th_ was found in the crystals of the pre-fusion form (that had p622 crystalline symmetry) [73]. 

Furthermore, a recent EM study performed on VSV revealed that the flat base of the virion is a privileged site for fusion [92] (Figure 3). The planar nature of this part of the virion could be propitious for the protein to assemble into a hexagonal p6 lattice. This local organization could favor the formation of the initial lipid intermediates (stalk or initial fusion pore) but also favor a concerted transition for the proteins making up the local network.

A second network of glycoproteins in their post-fusion conformation, in this case helical, can be observed on the cylindrical part of the VSV particles incubated below pH 6 [92]. When formed in the absence of a target membrane, this helical array disrupts the viral membrane suggesting that this network formation induces tension in the viral membrane. It has thus been proposed that these interactions between G glycoproteins, in their post-fusion conformation and located outside the contact zone, drive the enlargement of the initial fusion pore, which would have been formed in the contact zone between the flat base of the virion and the target membrane (Figure 3). 

### 5.5. Intermediates during the Conformational Change

During the conformational change, an intermediate conformation is formed with the fusion loops and the TM segment at the opposite ends of the molecule. In this conformation, the fusion loops are distal to the viral membrane and can easily interact with the target membrane. For RABV, this intermediate activated state is stabilized at low temperature and is formed early during the fusion process as demonstrated by hydrophobic photolabeling [60]. This type of intermediate is common for fusion proteins and is often referred to as the extended intermediate conformation [94].

**Figure 4:**
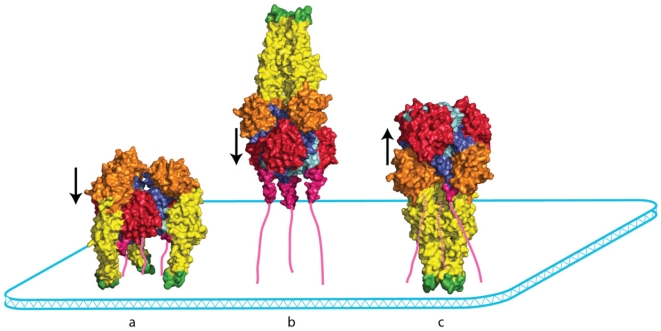
The topological problem associated with VSV G conformational changes. The VSV G pre- and post-fusion conformations are represented by a space-filling model viewed from the side. The C-terminal segments that reach the membrane are depicted as pink lines. In the pre-fusion conformation (**a**) and in a putative trimeric intermediate (**b**), the three fusion domains are located outside a pyramidal volume where the base would be the membrane and the sides would be defined by the ectodomain C-terminal segments. The converse occurs in the post-fusion state (**c**). Going from the pre-fusion trimer to the post‑fusion trimer is very complicated, if not impossible, without monomerization. The arrows indicate the orientation of the central helices in each conformation.

Complex topological issues exist for the structural transition pathway [51]. The initial steps leading to exposure of the fusion loops may maintain strict three-fold symmetry; however, this symmetry is disrupted by the refolding of the C-terminal portion of the molecule [95]. Furthermore, in the pre‑fusion state as well as in a putative extended trimeric intermediate conformation, the fusion domains are located outside the pyramidal volume defined by the viral membrane and the three C‑terminal segment whereas in the post-fusion state, they are located inside (Figure 4). If the molecule remains trimeric, it must perform very complex gymnastics. A much more plausible pathway would proceed through transient monomeric states of G [51]. Consistent with this view are the large differences between the trimeric interfaces of the pre- and post-fusion structure [73] as well as the fact that the VSV G trimers are unstable after detergent solubilization [4].

## 6. Cellular Biology of Rhabdovirus Entry into the Host Cell

### 6.1. Cellular Aspects of VSV Entry

**Figure 5:**
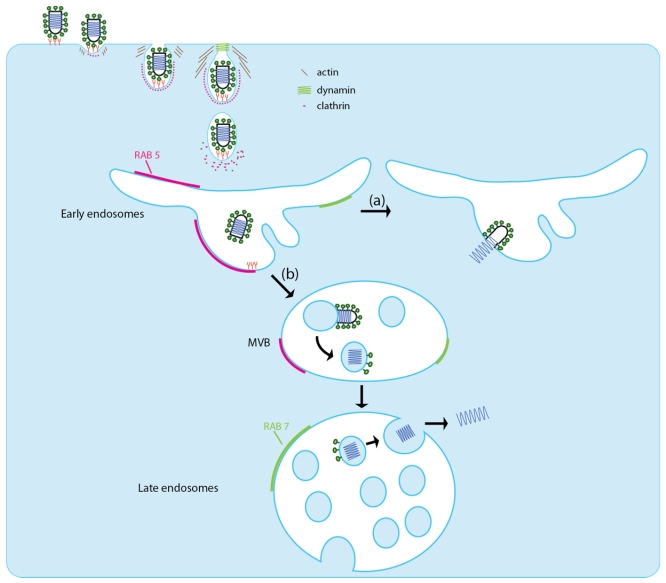
The two proposed pathways for VSV entry into the host cell. VSV binds to the host cell via an interaction with one or several unknown receptors. The VSV particles are then endocytosed in a clathrin-based, dynamin-2-dependent manner. The endocytosis vesicle is partially coated with clathrin and requires actin polymerization for efficient viral entry. Pathway (**a**): fusion occurs rapidly in early endosome and leads to the release of the nucleocapsid into the cytoplasm. Pathway (**b**): VSV fuses first with an internal vesicle inside multivesicular bodies. The nucleocapsid is then released in an internal vesicle. In the late endosome, these vesicles deliver the nucleocapsid to the cytoplasm through a back‑fusion mechanism. Small GTPase RAB5 (pink) is mainly present on early endosomes, whereas RAB7 (light green) is present mostly in late endosomes.

After binding to the cell surface, rhabdoviruses enter the host cell via the endocytic pathway. The exact cellular mechanism, however, was largely unknown until recent significant reports enlightened our understanding of this crucial step. As for other viruses, it is clear that rhabdoviruses can activate cellular signaling processes to trigger their own endocytosis and prepare the cell for invasion [96] (Figure 5). 

These studies have confirmed that the majority of VSV particles are endocytosed in a clathrin‑based, dynamin-2-dependent manner [97], which is consistent with previous electron microscopy studies showing VSV particles located within coated pits and coated vesicles [98]. Indeed, the entry of VSV into the host cell is sensitive to siRNA targeting the clathrin heavy chain [99], to chlorpromazine (a drug blocking clathrin-mediated endocytosis) [99] and to dynasore (an inhibitor of the GTPase activity of dynamin) [97]. Endocytosis of VSV is a fast process with a half-life of 2.5 to 3 minutes [97], and it appears that the virions are able to induce *de novo* formation of clathrin coated pits for their uptake [100]. 

Remarkably, VSV is significantly larger than the dimensions of a typical clathrin-coated vesicle. On average, virus-containing vesicles contain more clathrin and clathrin adaptor molecules than classical vesicles, but this increase is insufficient to permit full coating of the vesicle [100]. In fact, the vesicles used by the virus for entry are only partially clathrin-coated. The clathrin structures that internalize VSV require actin polymerization for efficient uptake into cells and treatment of cells with either cytochalasin D or latrunculin B inhibits entry by blocking the transition of virus-containing pits to the completed vesicles [100]. The requirement of local actin assembly for efficient entry is due to the length of the viral particle as the entry of shorter defective interfering particle (75 nm long *versus* 200 nm for VSV) does not depend on actin polymerization [101] (Figure 5). 

After endocytosis, fusion occurs rapidly within 1 to 2 minutes after internalization in early endosomes [97] (Figure 5, Pathway a). This is consistent with experiments using dominant negative forms of RAB GTPases that have shown that a RAB 5 mutant that blocks the maturation of endocytic vesicles to early endosomes inhibits VSV infection. RAB 7 mutant that blocks the traffic from the early to the late endosome and RAB 11 mutant that blocks the movement of the endocytosed cargo to recycling endosomes had no effect on VSV infection [102,103]. 

In contrast to the classical view, it has been proposed that the acid-activated fusion of VSV and the release of the nucleocapsid into the cytosol occur in two distinct steps [104]. The viral envelope would first fuse with an internal vesicle within the multivesicular bodies (MVB). The viral nucleocapsid would then be released into the cytosol by back-fusion of the internal vesicle with the limiting membrane of the MVB through the action of cellular fusion machinery (Figure 5 Pathway b). This view is supported by *in vitro* experiments that demonstrate that the phopholipidbis(monoacylglycero)phosphate (BMP) that is specific for the internal vesicles of MVB favors VSV fusion [69]. To date, most of the evidence obtained does not support this latter view but it remains possible that VSV can use different entry pathways depending on the cells and their physiological state.

### 6.2. RABV Entry into Neurons

**Figure 6:**
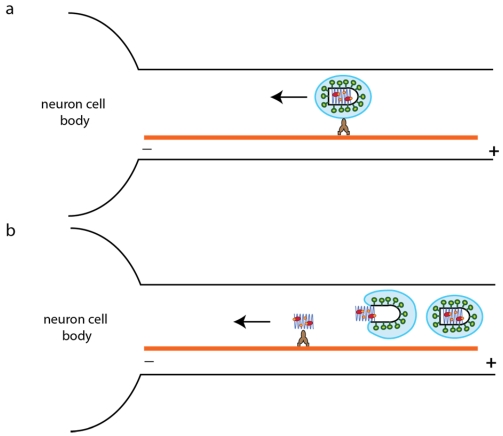
Two plausible pathways for rabies virus (RABV) retrograde transport in the axon. RABV may be transported by two different mechanisms on microtubules either in a vesicle (**a**) or as a free nucleocapsid (after membrane fusion) (**b**). As mentioned in the text, experimental data are in favor of pathway (a).

A rabies viral infection occurs at the nervous system periphery after an animal bite. The virus is then rapidly transported to the central nervous system. Two different pathways have been proposed for the transport of RV through the axon to the cell body where transcription and replication take place: Either fusion occurs early and the nucleocapsid is transported alone or the whole virion is transported inside a vesicle (Figure 6). 

It has been demonstrated that lyssavirus P proteins interact with dynein light chain LC8, a component of the dynein motor complex [105,106,107] suggesting that the ribonucleocapsid is transported by the cytoplasmic dynein motor complex. Nevertheless, as deletion of the LC8-binding site in the phosphoprotein does not affect viral transport from a peripheral site to the CNS, the contribution of dynein to the transport of viral RNP complexes has been questioned [108,109]. Furthermore, retroviruses pseudotyped with RABV G are transported in a retrograde way and reach the central nervous system after peripheral delivery in a manner similar to RABV [110,111]. This suggests that long distance transport along axons depends on RV G and that the whole virion is transported inside a vesicle (Figure 6a). This hypothesis has been challenged by infecting *in vitro*-differentiated NS20Y neuroblastoma cells with double-labeled virus particles that were composed of a red fluorescent envelope and a green fluorescent phosphoprotein. These experiments indicated that enveloped RV particles are transported as cargo inside vesicles by typical retrograde axonal transport [112]. 

### 6.3. Early Post Entry Events

Very little is known about the events, immediately following the release of the nucleocapsid into the cytoplasm that precedes and likely triggers primary transcription. Recently, it has been proposed that low-pH conformational changes in the G protein promote acidification of the virus interior, which facilitates the release of M from the ribonucleoprotein particles during uncoating [113] and is agreement with previous observations that G is able to induce pore formation in the viral membrane at low pH [114]. 

Following release of the nucleocapsid in the cytoplasm, RABV hijacks cellular machineries to induce the formation of inclusion bodies (similar to the Negri bodies found in the cytoplasm of infected neurons) [115,116]. These structures are detected very early after infection and constitute the sites of viral transcription and replication [116]. This observation is specific to RABV. In the case of VSV, primary transcription is not confined to any particular region of the cytoplasm [117]. Nevertheless, after viral protein synthesis, inclusion bodies are also observed that contain the viral RNA synthesis machinery and become the predominant sites of mRNA synthesis in the cell [118].

## 7. Conclusions

The determination of the pre- and post-fusion structures of VSV G, in addition to electron microscopy studies, have shed light on the molecular mechanism of the rhabdovirus fusion machinery. Nevertheless, the structures of intermediate conformations during the structural transition, and how they interact with and deform the fusing membranes, are still very elusive. Understanding the structure of full length intermediates (*i.e.*, with their TM domain) in association with membranes is now a major challenge in the field. 

Finally, as mentioned in the introduction, rhabdoviruses constitute a very wide family of viruses. Except for VSV and RABV, their cellular biology is largely unknown and, even for these two viruses, many questions remain unanswered. As an example, a recent human genome-wide siRNA screen identified 72 host genes required for VSV infection [119]. Some of the products of these genes, such as the solute carrier family 46 member 1 (SLC46A1: Proton-coupled folate transporter), seem to be involved in VSV entry and/or uncoating but their precise role at this stage is not known. In the near future, progress in super resolution microscopy together with the design of new recombinant fluorescent viruses (allowing direct observation of virions in live cells), should give an integrated view of the entry pathway of rhabdoviruses into their host cells. 
